# Prospective comparison of novel dark blood late gadolinium enhancement with conventional bright blood imaging for the detection of scar

**DOI:** 10.1186/s12968-017-0407-x

**Published:** 2017-11-21

**Authors:** Rohin Francis, Peter Kellman, Tushar Kotecha, Andrea Baggiano, Karl Norrington, Ana Martinez-Naharro, Sabrina Nordin, Daniel S. Knight, Roby D. Rakhit, Tim Lockie, Philip N. Hawkins, James C. Moon, Derek J. Hausenloy, Hui Xue, Michael S. Hansen, Marianna Fontana

**Affiliations:** 1 Cardiac MRI Unit, Royal Free Hospital, University College London, Rowland Hill Street, London, NW3 2PF UK; 2 Hatter Cardiovascular Institute, University College London, London, UK; 3 National Heart, Lung and Blood Institute, National Institutes of health, Bethesda, Maryland USA; 4 National Amyloidosis Centre, University College London, Royal Free Campus, London, UK; 5 Department of Cardiology, Royal Free Hospital, London, UK; 6 Barts Heart Centre, St. Bartholomew’s Hospital, London, UK; 7 Cardiovascular and Metabolic Disorders Program, Duke-National University of Singapore Medical School, Singapore, Singapore; 8 National Heart Research Institute Singapore, National Heart Centre, Singapore, Singapore; 9 Yong Loo Lin School of Medicine, National University Singapore, Singapore, Singapore; 10 The National Institute of Health Research University College London Hospitals Biomedical Research Centre, London, UK

**Keywords:** Late gadolinium enhancement, Myocardial infarction, Inversion recovery, Bright blood, Dark blood, PSIR

## Abstract

**Background:**

Conventional bright blood late gadolinium enhancement (bright blood LGE) imaging is a routine cardiovascular magnetic resonance (CMR) technique offering excellent contrast between areas of LGE and normal myocardium. However, contrast between LGE and blood is frequently poor. Dark blood LGE (DB LGE) employs an inversion recovery T2 preparation to suppress the blood pool, thereby increasing the contrast between the endocardium and blood. The objective of this study is to compare the diagnostic utility of a novel DB phase sensitive inversion recovery (PSIR) LGE CMR sequence to standard bright blood PSIR LGE.

**Methods:**

One hundred seventy-two patients referred for clinical CMR were scanned. A full left ventricle short axis stack was performed using both techniques, varying which was performed first in a 1:1 ratio. Two experienced observers analyzed all bright blood LGE and DB LGE stacks, which were randomized and anonymized. A scoring system was devised to quantify the presence and extent of gadolinium enhancement and the confidence with which the diagnosis could be made.

**Results:**

A total of 2752 LV segments were analyzed. There was very good inter-observer correlation for quantifying LGE. DB LGE analysis found 41.5% more segments that exhibited hyperenhancement in comparison to bright blood LGE (248/2752 segments (9.0%) positive for LGE with bright blood; 351/2752 segments (12.8%) positive for LGE with DB; *p* < 0.05). DB LGE also allowed observers to be more confident when diagnosing LGE (bright blood LGE high confidence in 154/248 regions (62.1%); DB LGE in 275/324 (84.9%) regions (p < 0.05)). Eighteen patients with no bright blood LGE were found to have had DB LGE, 15 of whom had no known history of myocardial infarction.

**Conclusions:**

DB LGE significantly increases LGE detection compared to standard bright blood LGE. It also increases observer confidence, particularly for subendocardial LGE, which may have important clinical implications.

## Background

Conventional bright blood late gadolinium enhancement (LGE) cardiovascular magnetic resonance (CMR) imaging offers excellent contrast between areas of bright LGE and normal myocardium, which is dark. However, contrast between myocardial gadolinium and the bright blood pool is frequently poor, meaning subendocardial LGE may be missed [1]. Infarct size is a key determinant of prognosis following a myocardial infarction (MI) [2] and a reduction in CMR-derived infarct size is a common surrogate endpoint in clinical trials [3, 4] and can guide therapeutic decisions [5]. The presence and characteristics of LGE in non-ischemic disease processes, such as hypertrophic cardiomyopathy, dilated cardiomyopathy or cardiac amyloidosis, also confer important diagnostic and prognostic information [6–8]. Detecting the presence and extent of LGE is therefore of major importance.

A large proportion of MIs are sub-endocardial and thus adjacent to the blood pool. The contrast between the blood and the MI in the inversion recovery (IR) image depends on variables such as contrast agent dosage, time from gadolinium administration, clearance rate and imaging parameters. Blood velocity may also have a role in the contrast, even though non-slice-selective IR is used. Therefore, as a result of mechanisms that are not fully characterized or controlled, it is not infrequent that subendocardial MIs are difficult to detect or clearly delineate.

Imaging at a later time point can result in better blood pool contrast for some subjects, but it may not be practical from the standpoint of clinical workflow to delay imaging, and in some instances contrast may worsen. Technical solutions to this problem are to use multiple contrasts such as T1 and T2 [1, 9], or to use blood suppression techniques [10–15]. The T2 of blood (250 ms) is significantly longer than that of myocardium (45 ms) and may be used to discriminate the MI from blood pool. In the “Multi-contrast delayed enhancement” (MCODE) approach [1, 16, 17], both T1- and T2-weighted images are acquired within the same breath-hold. The T1-weighted image is a phase sensitive inversion recovery (PSIR) image, and the T2-weighted image uses a radiofrequency (RF) preparation for T2-weighting. Both images are acquired at the same diastolic cardiac phase and are spatially registered facilitating fusion of the images to enhance the subendocardial border.

An alternative approach that also exploits the difference in tissue T1 and T2 is to use a segmented cine IR with balanced steady state free precession (bSSFP) readout [9]. In this method, each cardiac phase has a unique T1 and T2 contrast as determined by both the inversion recovery and the bSSFP readout which has a √(T2/T1) steady state dependence. The blood will appear dark at a different cardiac phase than the MI. In an alternative approach an IR fast low angle shot (FLASH) sequence may be modified to null both the blood and the normal myocardium using 2 inversions with carefully chosen inversion times [10, 18]. These methods achieve suppression of blood at the price of signal-to-noise ratio (SNR) and largely rely on the T1 of scar being shorter than blood. It has recently been shown that PSIR imaging with a shorter inversion time set to null the blood may also be used to improve the contrast between the MI and adjacent blood pool signal without requiring a special RF preparation [19].

Alternatively, LGE with blood suppression may be achieved my combining either a T2 preparation [12, 14] with IR or by combining a magnetization transfer (MT) preparation with IR [11, 13]. In these schemes, the myocardial signal is reduced relative to the blood signal thereby reducing the inversion time to null the myocardium. In this way, it is possible to null both the myocardium and the blood at the same time. The order of the T2 and IR preparations may be applied as T2-IR [12] or IR-T2 [14]. Both of these previously reported schemes used a FLASH readout. In this present work [15], we combined an IR-T2 with a single shot bSSFP readout and respiratory motion corrected averaging [16, 20, 21] to achieve the acceptable SNR while maintaining the desired spatial and temporal resolution. In this manner, imaging is conducted free-breathing which has benefits for image quality, patient comfort, and clinical workflow in both adults [22] and children [23]. Furthermore, by using a PSIR reconstruction [24] the blood signal may be made darker than the myocardium (i.e., negative signal values) thereby providing contrast between the blood and both the MI and remote myocardium [15].

Free-breathing, motion-corrected (MOCO) bright blood LGE has been demonstrated to be comparable to breath-held sequences and offers particular advantages in terms of patient comfort, image quality and clinical workflow [22]. This study sought to determine whether DB LGE could detect more scar and/or increase both inter-observer variability, compared to conventional PSIR LGE imaging. CMR imaging, initially validated against histology in the seminal work done by Kim et al., has become a widely accepted component of a standard clinical scan [25] although most institutions are not using the specific imaging protocol that was validated [26]. As the clinical utility of bright blood LGE has widened, a degree of variation in the technique’s application has been introduced; such as lower gadolinium dose, MOCO, new image reconstruction techniques such PSIR, and the proposed DB LGE, which although not histologically validated, have been shown to provide clear benefits.

## Methods

A total of 172 adult patients referred for clinical CMR were scanned using a 1.5 T scanner (Magnetom Aera, Siemens Healthineers, Erlangen, Germany). All ethics were approved by the local ethics committee and all participants provided written informed consent.

Indications for CMR were a typical case-mix for a clinical CMR center. After standard clinical scan sequences (pilots, transverse white and black blood images, cines images to assess left ventricular volumes and mass), a full short axis stack of bright blood LGE and DB LGE images were acquired approximately between 8 and 15 min after the administration of contrast agent (Gadoterate meglumine, 0.1 mmol/kg). This dose reflects local protocol, which is the current clinical standard in other centers as well [8, 27–30]. All LGE imaging was acquired free-breathing with MOCO and was reconstructed using PSIR. Patients were assigned to undergo either first receive bright blood LGE or DB LGE sequences in a 1:1 ratio. The duration of the acquisition for the bright blood LGE approach was 16 heart beats per slice (8 measurements × 2 RR) and for the DB LGE approach was 32 heart beats (16 measurements), with an additional T1 map for determination of inversion and T2 preparation time parameters [15].

### LGE image acquisition

Both DB LGE and bright blood PSIR LGE images were acquired free-breathing using a single shot bSSFP sequence with MOCO averaging of repeated measurements [15, 16]. The single-shot bSSFP sequence, rather than breath-held segmented gradient echo (GRE) was used so that PSIR bright blood LGE would be free-breathing for both protocols being compared. Free-breathing LGE is becoming widely used to avoid artifacts due to poor breath-holding and irregular rhythm, and is used by default at our institution on all patients.

**Fig. 1:**
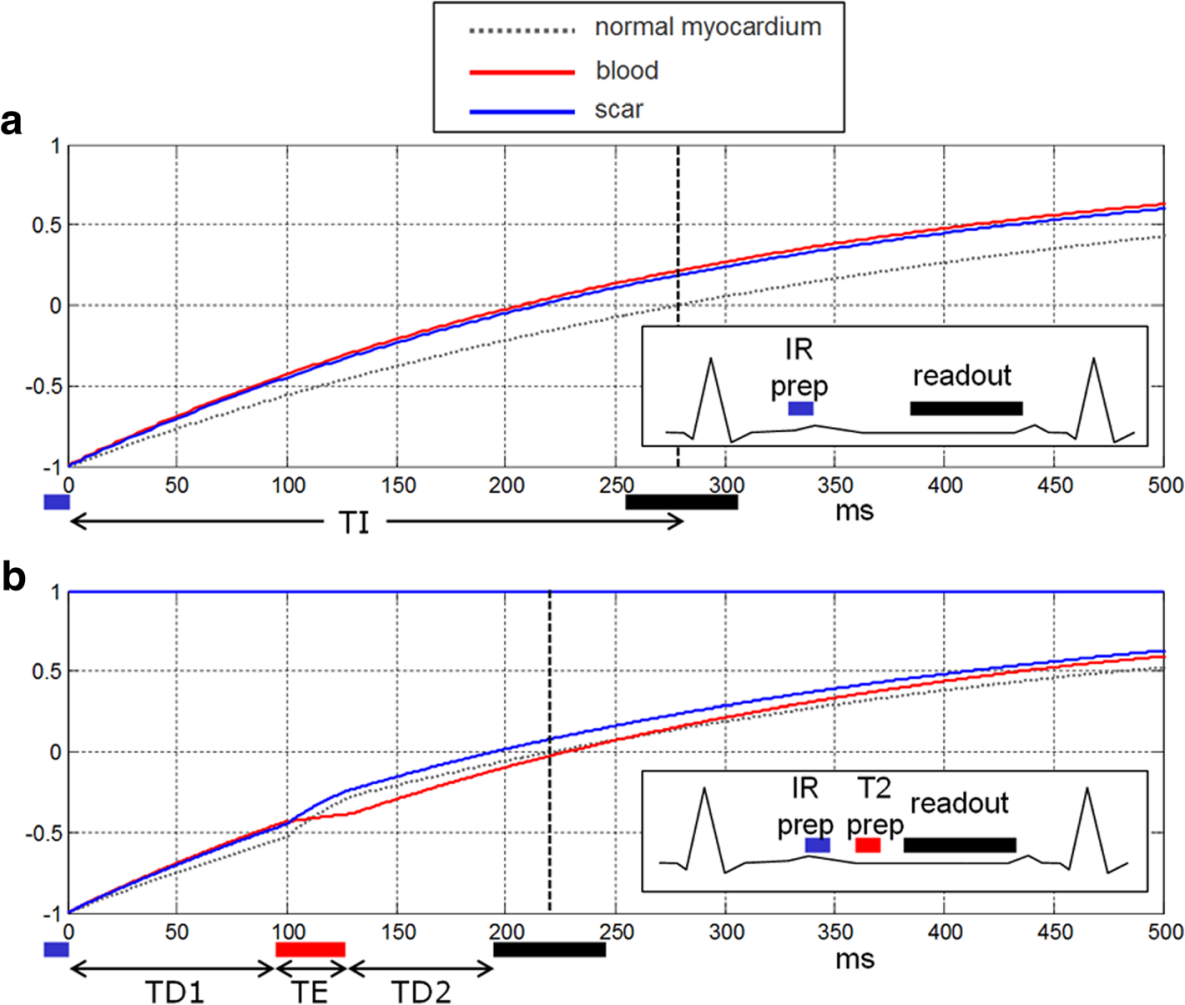
**a** inversion recovery (IR) for bright blood LGE in case with scar signal (blue) less than blood (red) resulting in poor contrast. **b** IR for dark blood (DB) late gadolinium enhancement (LGE) using combined IR and T2 preparation to shift the null time of blood relative to the normal myocardium. In this case the delays are chosen such that the blood signal (red) is less than the myocardium (dashed grey) resulting in dark blood using phase sensitive inversion recovery (PSIR) reconstruction, which preserves the signal polarity. Inversion times to null the normal myocardium are depicted by vertical dashed lines. The loss in signal-to-noise ratio (SNR) due to the T2 preparation is mitigated by increased respiratory motion corrected averaging (MOCO) [15]

DB LGE images were acquired with parameters matched to the bright blood LGE images except for the TI. DB LGE images were implemented by adding a T2 prep between the IR preparation and the readout. This shifted the null time of the myocardium relative to blood making it possible to choose delays that simultaneously null both myocardium and blood, with the aim to achieve a positive contrast between scar and both blood and normal myocardium. A simplified diagram of the sequence timing and illustration of the inversion recovery is shown in Fig. 1 for bright blood LGE and DB LGE sequences. The DB LGE acquisition technique has been recently described in detail [15]. Briefly, the myocardial and blood T1 were measured using a long axis T1-mapping scout scan using the MOdified Look-Locker Inversion recovery (MOLLI) approach with a modified protocol using MOCO and a 4s(1s)3s(1s)2s acquisition scheme that was acquired in 11 s [31]. Blood and myocardial T1 values were measured by the operator and entered on the scanner in the user interface (UI) [15]. The blood suppression was specified as a fixed default value (delta) which was darker than the myocardium when the value delta <0. The sequence calculated preparation delays (TD1, TD2) and T2 preparation echo time (TE) (illustrated in Fig. 1) using a strategy that sought to achieve the desired blood suppression with the shortest TE so as to minimize SNR loss.

Respiratory MOCO averaging was used for both bright blood LGE and DB LGE protocols. The number of averages was double for the DB LGE protocol as compared to the bright blood LGE protocol to improve the contrast-to noise-ratio (CNR) by √2, and thereby mitigate the SNR loss due to the T2 weighting. It was previously shown that by using twice the number of averages the DB LGE achieved approximately the same SNR [15] as the bright blood LGE**.** In order to mitigate through-plane motion during the free-breathing acquisition (both bright and DB), 50% of the acquired measurements are discarded [20]. This strategy has been demonstrated to work well for both short- and long-axis orientations. Measurements are discarded based on a mean squared error similarity criteria.

By choosing the delta parameter less than 0, the blood becomes negative relative to the myocardium and will appear blacker when using a phase sensitive reconstruction [15]. In this case, if the DB LGE image is window leveled to display the normal myocardium nulled, then the blood will be displayed darker and there will be no apparent contrast between the myocardium and adjacent myocardium. For this reason, the window level is adjusted to make the normal myocardium slightly grey such that the endocardial border with the blood pool is clearly delineated.

With these protocols (Table 1), a stack of 10 slices is acquired in 160 heart beats (bright blood) and 320 heart beats (dark blood), corresponding to 2:20 min and 4:35 min at 70 bpm, respectively. During this time the gadolinium is clearing, thus the inversion time to null the normal myocardium is slightly increasing. This would result in a loss of contrast when using magnitude IR reconstruction requiring continued readjustment of the inversion time (TI) [26], however using PSIR, which is insensitive to inversion time [24] it is not necessary to adjust the TI. This facilitates scanning the entire stack in a single acquisition and simplifies the workflow.

**Table 1:** Imaging protocol parameters

	Bright Blood LGE	DB LGE
Preparation	Inversion Preparation	Inversion Preparation & T2 preparation
Readout	Single shot, bSSFP (FA_IR_ = 50°, FA _PD_ = 8°)	
Typical FOV / resolution	360 × 270 mm^2^ 1.4 × 1.9 × 8 mm^3^	
Matrix size	256 × 144 (parallel imaging factor 2)	
Number of acquired measurements	8	16
T2 preparation TE	10–40 ms	
TE/TR	1.2/2.8 ms	
ECG triggering	Inversions every 2 RR (HR < 90 bpm) Inversions every 3 RR (HR > 90 bpm)	

*DB* dark blood, *ECG* electrocardiom, *FOV* field-of-view, *LGE* late gadolinium enhancement, *TE* echo time, *TR* repetition time

The imaging parameters for the DB LGE were saved in the raw data which was stored for follow-up analysis. Mean and standard deviation (SD) of the measured T1 for myocardium and blood from the T1-map scout scan and values for TD1, TD2, and TE calculated by the sequence were calculated for all DB LGE scans during this reporting period.

This bright blood LGE protocol used in this study is now widely used at a number of institutions. We chose to compare the DB LGE protocol with 2× averaging (16 vs 8 measurements) in order to match CNR of MI to normal myocardium [15], as described above. A small sub-study was conducted (*n* = 21 subjects) to compare the effect of averaging and spatial resolution on detectability of scar. A single slice was imaged using 4 protocols in rapid succession: a) bright blood LGE with 8 measurements, b) bright blood LGE with 16 measurements to equal the DB LGE, c) a higher spatial resolution (288 × 180) bright blood LGE with 16 measurements (approx. same CNR as DB LGE), and d) DB LGE with 16 measurements. In cases for which the MI signal is equal or less than the blood signal, it was hypothesized that the increased averaging or spatial resolution would not improve the scar contrast. CNR was measured between MI and remote, MI and blood, and blood and remote for all 4 protocols compared. CNR was measured by difference between measured SNR values with SNR determined by previously validated SNR scaled reconstruction which accounted for all parallel imaging losses [15, 32].

### Image analysis

Image analysis was performed by two independent observers (RF and TK). The bright blood LGE and DB LGE stacks were separated and evaluated in a random anonymized order using open-source software (Osirix; the Osirix Foundation, Geneva, Switzerland) [33]. A qualitative rather than quantitative approach was used to score the LGE since quantitative methods manually delineate the blood boundary and are not developed or validated for dark blood methods.

### LGE assessment

The full left ventricular short-axis stack for each technique was analyzed on a per-segment basis using the American Heart Association model [34], excluding the apical cap. Cine images were not available to the observers but a static 4-chamber image was provided to allow localization of short axis slices.

The transmural extent of LGE was evaluated using a three-point Likert score (0, none; 1, <50% wall thickness LGE; 2, >50% wall thickness LGE). This allowed quantification of not only how many segments were affected but using the total from the 16 LV segments, an additional measure of LV ‘scar burden’, with a theoretical maximum score of 32. In addition, the level of certainty was rated on a per-segment basis using a binary scale (confident/non-confident).

### Statistical analysis

Statistical analysis was performed using SPSS Statistics Version 24 (International Business Machines, Armonk, New York, USA). Inter-observer variability was assessed by calculating the intraclass correlation coefficient for each parameter assessed. The chi-square test or Fisher exact test was used to compare results between both imaging techniques. The change in the number of segments identified using both techniques was analyzed using a paired t-test.

Each patient was analyzed for the comparison as a single data point (each patient had a total score for each imaging modality going from 0 to a theoretical maximum of 16). This approach was used to account for the fact that segments within the same patient are not independent. Statistical significance was defined as *p* < 0.05.

## Results

Of 176 recruited patients, four were excluded from analysis (Table 2) including three due to poor LGE image quality (1 bright blood LGE and 2 DB LGE caused by complete gadolinium washout; these 3 cases had been allocated to be performed second) and one patient terminated their scan before completion. The remaining 172 patients were analysed using both techniques, corresponding to a total of 2752 LV segments.

**Table 2:** Baseline characteristics of patient population (*n* = 172). Values are n, mean ± standard deviation (SD), or frequency (%)

Age (years)	59 ± 15
Gender (male (%))	101 (58.7)
Weight (kg)	76 ± 14
Height (cm)	171 ± 10
Body Surface area (m^2^)	2.0 ± 0.21
Prior myocardial infarction (%)	46 (26.7)
Prior percutaneous coronary intervention (%)	35 (20.3)
Prior coronary artery bypass surgery (%)	6 (3.5)
CMR parameters	
LVEDVi, mL/m^2^	81 ± 26
LVESVi, mL/m^2^	38 ± 30
SVi, mL/m^2^	42 ± 12
LAAi, cm^2/^m^2^	12 ± 3
RAAi, cm^2^/m^2^	11 ± 3
Mitral regurgitation (by CMR)	
Not present	134 (77.9%)
Mild	27 (15.7%)
Moderate	9 (5.2%)
Severe	2 (1.2%)

*CMR* cardiovascular magnetic resonance, *LVEDVi* left ventricular end-diastolic volume index, *LVESVI* left ventricular end-systolic volume index, *LAAi* left atrial appendage area index, *RAAi* right atrial appendage area index, *SVI* stroke volume index

Half the patients (*n* = 86) underwent bright blood LGE first and the other half underwent sequenced LGE first. The time following contrast administration for each stack was comparable across the two groups (Table 3).

**Table 3:** Average (mean ± standard deviation (SD)) time of initiating each short axis stack, recorded as minutes and seconds after administration of gadolinium. Patients were split into two equal groups, one undergoing bright blood LGE first, the other undergoing DB LGE first

	Bright blood LGE first	DB LGE first
Time of first stack (min:sec)	09:04 (±3:26)	09:49 (±3:06)
Time of second stack (min:sec)	16:33 (±3:47)	16:00 (±3.48)

*DB* dark blood, *LGE* late gadolinium enhancement

### Inter-observer agreement and confidence

There was high inter-observer agreement for both sequences when determining the presence of LGE as well as assessing high confidence (Table 4).

**Table 4:** Inter-observer agreement for presence of LGE (number of segments showing LGE) and high LGE diagnostic confidence, for both bright blood and DB sequences

	Bright Blood LGE	DB LGE
	ICC	ICC
Presence of LGE	0.88 (0.84–0.91)	0.92 (0.89–0.94)
High confidence LGE	0.75 (0.70–0.81)	0.89 (0.86–0.92)

Using bright blood LGE, hyperenhancement could be diagnosed with high confidence in 154/248 regions (62.1%) and low confidence in the remainder (*n* = 94/248; 37.9%). With DB LGE, hyperenhancement was diagnosed with high confidence in 275/324 (84.9%) regions and low confidence in the remainder (*n* = 49/324; 15.1%); DB LGE allowed a significantly higher proportion of LGE-containing regions to be assigned high confidence (*p* < 0.05) (Table 5).

**Table 5:** Comparison of LV LGE detection, transmurality, diagnostic confidence and mean LGE burden between bright blood and DB imaging techniques

	Bright Blood LGE	DB LGE
Segments with LGE	248/2752 (9.0%)	351/2752 (12.8%)
> 50% transmurality	126 (50.8%)	169 (48.1%)
< 50% transmurality	122 (49.2%)	182 (51.9%)
LGE with high confidence	154/248 (62.1%)	302/351 (86.0%)
LGE with low confidence	94/248 (37.9%)	49/351 (14.0%)
Average LV LGE burden	2.23	2.94

### LGE in LV segments

**Fig. 2:**
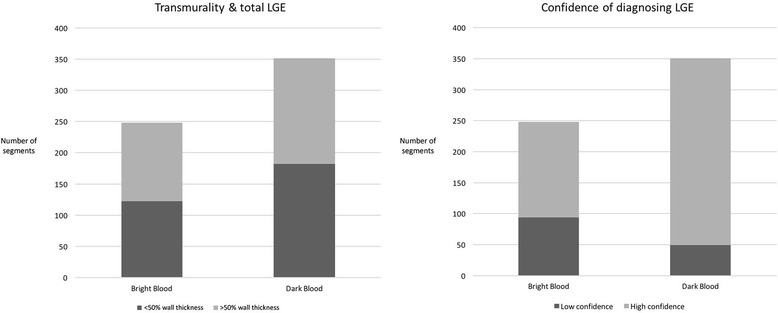
Total number of left ventricular (LV) segments identified (left) and diagnostic confidence when diagnosing late gadolinium enhancement (LGE) (right) using both bright blood and dark blood (DB) imaging. The time after gadolinium administration is recorded for each sequence (minutes:seconds)

DB LGE identified significantly more areas of hyperenhancement than bright blood LGE (Figs. 1 and 2). DB LGE analysis found 41.5% more segments that exhibited LGE in comparison to bright blood LGE (248/2752 segments (9.0%) positive for hyperenhancement with bright blood; 351/2752 segments (12.8%) positive for LGE with DB LGE; p < 0.05).

On a per-patient basis, bright blood LGE identified definite (high confidence) hyperenhancement in 58 patients, whereas the DB LGE allowed detection of hyperenhancement in 81 patients. The average total hyperenhancement burden per patient was scored 31.8% higher using DB LGE. Eighteen patients assessed to have no hyperenhancement with bright blood LGE were found to have hyperenhancement using DB LGE. Of these, 15 did not have a past history of MI, 4 of whom had been admitted with a troponin-positive acute coronary event but had no significant coronary artery disease on invasive angiography.

The majority of segments containing hyperenhancement identified by either sequence were subendocardially distributed, however some patients exhibited mid-wall and epicardial hyperenhancement. Epicardial hyperenhancement was seen in 5/2752 segments using both techniques. Mid-wall hyperenhancement was seen in 9/2752 segments with bright blood LGE and 6/2752 using the DB LGE.

Eleven patients (23 segments) assessed to have low confidence scar with bright blood LGE were found to be free of hyperenhancement on DB LGE. Thirteen patients (33 segments) assessed as demonstrating at least one segment of <50% wall thickness hyperenhancement were upgraded to >50% with DB LGE. A total of 103 segments were reclassified from normal to showing scar, all of which was subendocardial in distribution. However, 3 patients with definite (high confidence) hyperenhancement on bright blood failed to show hyperenhancement on DB LGE, all of whom had non-sub-endocardial hyperenhancement distribution.

### LGE in the papillary muscles

**Fig. 3:**
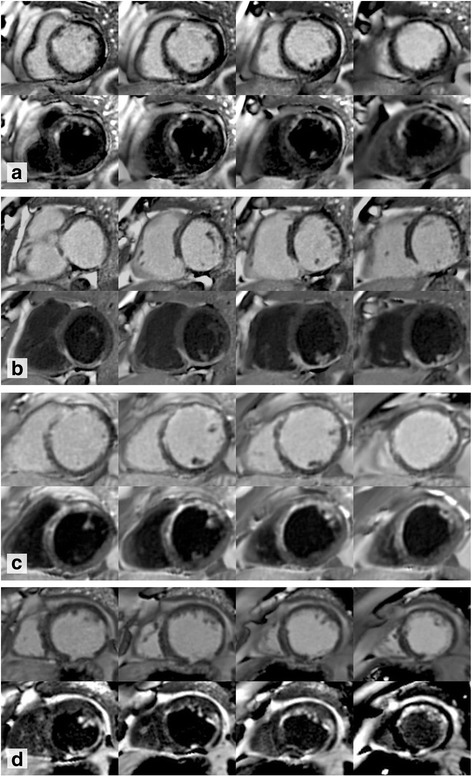
Consecutive short axis slices from four patients. In each panel, bright blood late gadolinium enhancement (LGE) sequences appear above dark blood (DB) LGE. The time after gadolinium administration is recorded for each sequence (minutes:seconds). **Patient A:** DB LGE reveals a large left anterior descending (LAD) territory infarct, the size and borders of which are not clearly delineated on bright blood imaging. Bright blood 14:43, DB 21:21. **Patient B:** Inferior infarction can be seen on the bright blood images but it is difficult to appreciate if the basal slice is affected or to see the endomyocardial border at all. DB images reveal segments showing LGE and the myocardial structure. Bright blood 10:31, DB 16:07. **Patient C:** Bright blood images suggest anterior LGE but it is difficult to quantify accurately. DB LGE shows the extent of an anterior infarct. Bright blood 8:13, DB 16:14. **Patient D:** Poor contrast between scar and the blood pool masks antero-lateral scar, seen easily with DB LGE. Bright 6:59, DB 12:12

**Fig. 4:**
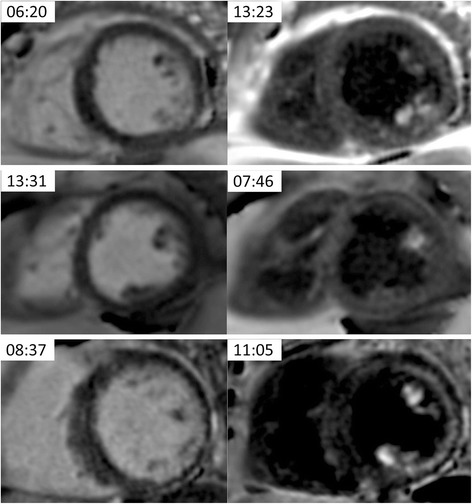
Paired SA slices from 3 patients showing papillary muscle late gadolinium enhancement (LGE). In the top left corner of each image is the time after administration of gadolinium (minutes:seconds)

Using bright blood LGE, only 5 patients were thought to have hyperenhancement in either papillary muscle, however with DB LGE, 33 patients (19%) were identified with papillary muscle hyperenhancement (Fig. 3). Of these, 9 had infarcted myocardium in segments adjacent to the affected papillary muscle but the remaining 26 appeared to have isolated papillary muscle hyperenhancement. Overall, there was no significant correlation to presence of papillary muscle LGE with mitral regurgitation severity nor indexed left atrial area (LAA) (Fig. 4).

### User parameters

The measured myocardial and blood T1 measured using the MOLLI scout prior to the DB LGE and entered as parameters to the sequence user interface were 505 ± 61 ms (mean ± SD) and 377 ± 65 ms, respectively. The values of derived parameters calculated by the sequence were: TE = 18.7 ± 6.2 ms, TD1 = 43.7 ± 30.2 ms, and TD2 = 60.4 ± 35.9 ms.

### Increased averaging

A sub-study examining the effect of averaging confirmed that increased averaging did not improve the apparent contrast of bright blood LGE in cases with poor blood pool contrast. Twenty-one patients were imaged, that were positive for LGE.

The CNR between the MI and adjacent blood pool was: 6.2 ± 10.5, 7.3 ± 14.0, 4.3 ± 8.7, and 26.3 ± 10.7 (mean ± SD), for bright blood LGE with 8 avg. (average), bright blood LGE with 16 avg., high resolution bright blood LGE with 16 avg., and DB LGEwith 16 avg., respectively. The CNR between the MI and remote myocardium was: 21.0 ± 10.7, 28.1 ± 16.2, 14.6 ± 9.5, and 17.9 ± 10.0 (mean ± SD), for bright blood LGE with 8 avg., bright blood LGE with 16 avg., high resolution bright blood LGE with 16 avg., and DB LGE with 16 avg. respectively.

The CNR for the DB LGE with 16 averages for this group was 15% less than the bright blood LGEusing 8 averages which is consistent with previous measurements [15]. The CNR between the remote myocardium and adjacent blood pool was: −14.8 ± 9.3, −20.8 ± 13.8, −10.3 ± 6.2, and +8.4 ± 5.9 (mean ± SD), for bright blood LGE with 8 avg., bright blood with 16 avg., high resolution bright blood LGE with 16 average, and DB LGE with 16 avg. respectively, providing positive contrast for the remote myocardium.

## Discussion

This prospective study shows that a dark blood LGE with MOCO approach can be implemented into a clinical workflow. It requires the acquisition of a single T1 map used to measure T1 blood and myocardium. The total time for the T1 map and the left ventricular short axis stack is approximately 4 min depending on the heart rate. The DB LGE detects more hyperenhancement overall, particularly subendocardial hyperenhancement. In 9% of patients, DB LGE detected missed infarcts that are likely of high clinical importance. We also show that clinicians found the DB LGE easier to interpret and that interpretations are associated with higher confidence.

Fifteen patients without a known MI diagnosis were declared free of any scar using bright blood LGE, but actually had subendocardial hyperenhancement on DB LGE. There is potential that this approach could lead to significant change in patient management. Four of these patients found to have hyperenhancement with only DB LGE had been admitted acutely for an acute coronary syndrome but had unobstructed coronary arteries on invasive angiography. A meta-analysis of patients with MI with non-obstructed coronary arteries showed that typical CMR features of infarct hyperenhancement are only seen in 24% of cases and no significant abnormality in 26% [35]. It is possible that some patients currently deemed free of MI on conventional bright blood LGE may in fact have typical subendocardial hyperenhancement caused by MI and should receive secondary prevention therapies. The other 11 patients out of the 15 with DB LGE but negative bright blood images were non-urgent patients referred for CMR for a variety of reasons. One patient was undergoing a surveillance CMR for hypertrophic cardiomyopathy and had never been diagnosed with any hyperenhancement previously, yet DB LGE revealed hyperenhancement, which may have prognostic relevance.

A major difference between the sequences is the marked difference in apparent papillary muscle hyperenhancement. Papillary muscles are comparatively small and surrounded by blood so normally very hard to distinguish hyperenhancement on conventional bright blood LGE, but more easily visible on DB LGE. Care was taken to avoid diagnosing papillary muscle hyperenhancement on a short-axis slice selected too close to the base as fibrous structures such as chordae enhance brightly. Aside from 9 patients who had papillary muscle LGE in association with a MI (5 inferior, 4 anterior), the majority of the additional patients found to have hyperenhancement using the DB LGE had enhancement limited to a papillary muscle. As papillary muscle hyperenhancement is rarely so easily seen, the clinical significance of isolated papillary muscle hyperenhancement is unclear. Studies examining the relevance of papillary muscle hyperenhancement on bright blood LGE have found 14% of patients with confirmed ST-segment elevation MIs (STEMIs) display papillary muscle hyperenhancement and that there is an association with mitral regurgitation [36]. In our cohort of 121 patients, we recorded 19.2% as positive for papillary muscle hyperenhancement on DB LGE and found no correlation with mitral regurgitation and indexed LAA, however only 11 patients had significant mitral regurgitation, so further investigation would be warranted. If the appearances are consistent with true LGE, these findings might suggest some degree of papillary muscle hyperenhancement is within normal limits and simply not previously detected with bright blood LGE.

In this paper a qualitative approach was used for scar detection. Although there are published methods for quantitative measurement of size, there is no consensus on these methods, moreover most are not widely available. Most importantly these methods have been developed for bright blood LGE and have not been tested on images obtained using a DB LGE.

Quantifying infarct size involves several challenges. One is detecting the subendocardial border and another is determining which myocardial pixels are scar. The current tools address the latter, either by setting a threshold relative to the noise (a fixed number of standard deviations) or using a full width half max (FWHM) to mitigate partial volume blurring. However, the published methods rely on manually traced endocardial borders. In cases with poor infarct-blood contrast, the subendocardial border is particularly difficult to measure. This demonstrates that the largest source of error in quantifying scar size will be this manually traced border. This is an area deserving of a study but is outside the scope of our current manuscript.

Some preliminary studies have used alternative methods to produce a dark blood pool and showed that these sequences are helpful for improved delineation of subendocardial infarction, particularly when the T1 values for enhancing myocardium and ventricular blood are similar, which is frequently the case. [10, 11] [12–14]. In this prospective study we use a new DB LGE approach in a relatively large patient cohort and performed a comparison with whole heart coverage between standard bright blood LGE images and novel DB LGE images. Furthermore, combining a PSIR-T2 preparation with a single shot bSSFP readout and respiratory motion corrected averaging [16, 20, 21], imaging acquisition is conducted free-breathing, which has benefits for image quality, patient comfort, and clinical workflow [15].

The use of cine images in conjunction with late enhancement images has been cited as a means of addressing the issue of subendocardial MI cases with low contrast between the MI and blood pool [26]. Cine images offer excellent resolution and contrast between blood and myocardium, and this technique is often very helpful, although it is sometimes time-consuming to interpret the results due to differences in spatial and temporal resolution. It is also noted that cine and LGE images are acquired at different times and may have different slice positions, even though the slice prescription is the same, due to differences in respiratory position. Even small differences may complicate the interpretation or make it impossible to resolve, since the subendocardial MI may be very narrow or small. The accuracy of the subendocardial border will affect the assessment of transmurality, an important prognostic indicator. This is not to imply that cine imaging is not useful or that one should not use all available data to achieve the best interpretation, but rather to stress potential pitfalls. The proposed DB LGE method provides an easy means of enhanced detection.

The DB LGE technique is robust in that it is fairly insensitive to the measured T1’s entered as input parameters [15]. By virtue of using PSIR reconstruction, errors in the measured myocardial T1 results in an imperfect null, which may be adjusted by window-level in the displayed PSIR image. Errors in the blood T1 result in a different degree of blood suppression. The current protocol sets the blood to be less than the myocardium thus appearing blacker in the PSIR image, and the parameter is set for adequate contrast so that typical variations are not noticeable.

Both the non-selective double IR, the IR-T2 and MT preparations achieve a large degree of flow independence. In the case of the IR-T2 approach, the blood suppression will depend on the T2 thus blood oxygenation will cause difference in blood between the LV and RV. The MT preparation is independent of T2 but will depend on the effective B1 (transmit flip angle) which will vary across the heart. The myocardial T2 is well characterized and therefore SNR losses due to T2 preparation may be predicted and compensated for by averaging. The myocardial signal loss due to MT is less well characterized, thus the performance is less well characterized. Similarly, methods that rely strictly on T1 differences are difficult to predict performance since there are wide variations in gadolinium concentrations due to a variety of mechanisms and timing which is difficult to control in a clinical environment.

### Limitations

We found increased LGE detection with DB LGE. We also noticed an increase in observer confidence, particularly for subendocardial hyperenhancement. Whilst the authors feel this to be related to improved scar detection, there was no histologic confirmation. Future studies validating histologically these findings would be of great value.

**Fig. 5:**
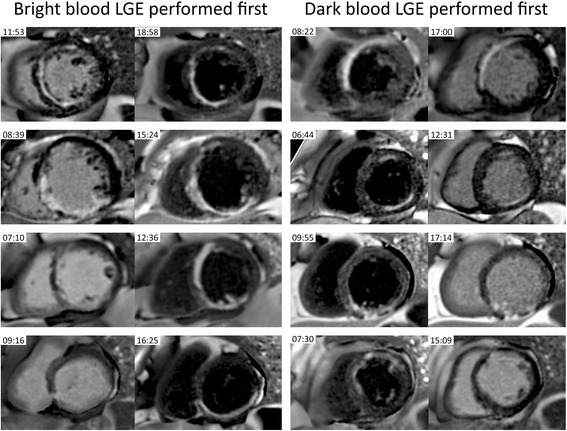
Examples of 4 patients that underwent bright blood late gadolinium enhancement (LGE) first and dark blood (DB) LGE second (left column) and 4 additional patients that underwent the sequences in the opposite order (right column). The bright blood LGE, for either acquisition order, exhibits a range of contrast between the subendocardial scar and adjacent blood pool, from good (top rows) to poor (bottom rows) contrast, whereas the DB LGE had excellent contrast in all cases. In the top left corner of each image is the time after administration of gadolinium (minutes:seconds)

DB LGE acquisition is prolonged as compared with bright blood LGE due to the increased number of measurements (16 vs 8). In our experience, a full left ventricular short axis stack required approximately 4 min. However, due to the fact the sequences are free-breathing, this does not significantly impact on patient comfort. A single gadolinium dose was examined. Single dose and 1.5 dose gadolinium is often cited and shown to provide excellent quality [26] although the original protocols used in widely cited validation experiments [25] used triple dose. Recent validation has been performed for 1.5 dose [37] whilst no validation studies are available for single dose in terms of a histological reference standard. There are recent dose comparison studies that indicate that single dose is a effective for LGE [28, 30]. The time from gadolinium injection for LGE imaging was described in the original LGE validation paper [25] at about 20 min for accurately delineating scar. In the present paper an 8–15 min window was selected. This is the result of the single gadolinium dose and is in line with the T1 mapping guidelines for extracellular volume mapping currently [31, 38]. These guidelines for mapping are believed to be broadly relevant to imaging fibrosis since the mechanism of gadolinium distribution dictates the LGE contrast. The DB LGE approach requires manual entry of myocardial and blood pool T1 in sequence UI [15], which are derived from a single post-contrast MOLLI map. DB LGE does require more manual windowing prior to analysis than bright blood LGE, but again this is straightforward after a small amount of practice. An appropriately windowed DB LGE image produces a black blood pool, dark grey myocardium and white gadolinium enhancement. Examples of patients undergoing bright blood LGE before DB LGE and vice versa are shown in Fig. 5.

Mid-wall, epicardial and right ventricular LGE diagnoses were noted in some patients, but the small numbers do not allow conclusions to be drawn and this represents an important area of further investigation. Given these limitations, at present DB LGE approach should be considered not as replacement for conventional LGE, but providing considerable added value. Further work is needed to explore the clinical role in different patient cohorts including both ischemic and non ischemic aetiologies.

## Conclusion

DB LGE is a novel technique to improve LGE detection by nulling the blood pool. DB LGE detects more areas of subendocardial hyperenhancement than conventional bright blood LGE and allows a much higher level of confidence when deciding if a region of myocardium exhibits any scar. It is likely to be of greatest use when looking for hyperenhancement at the endomyocardial-blood border and thus may be of particular use in patients with suspected or proven ischemic heart disease.

## Abbreviations

bSSFP: Balanced steady state free precession
CMR: Cardiovascular magnetic resonance imaging
CNR: Contrast to noise ratio
DB: Dark blood
GRE: Gradient echo
IR: Inversion recovery
LAA: Left atrial area
LGE: Late gadolinium enhancement
MI: Myocardial infarction
MOCO: (respiratory) Motion-corrected
MOLLI: MOdified Look-Locker Inversion Recovery
MT: Magnetization transfer
PSIR: Phase sensitive inversion recovery
RF: Radiofrequency
SD: Standard deviation
SNR: Signal to noise ratio
